# Leguminous green manure intercropping changes the soil microbial community and increases soil nutrients and key quality components of tea leaves

**DOI:** 10.1093/hr/uhae018

**Published:** 2024-01-17

**Authors:** Yu Duan, Ting Wang, Xiaogang Lei, Yu Cao, Lefeng Liu, Zhongwei Zou, Yuanchun Ma, Xujun Zhu, Wanping Fang

**Affiliations:** College of Horticulture, Nanjing Agricultural University, Nanjing 210095, China; College of Horticulture, Nanjing Agricultural University, Nanjing 210095, China; College of Horticulture, Nanjing Agricultural University, Nanjing 210095, China; College of Horticulture, Nanjing Agricultural University, Nanjing 210095, China; College of Horticulture, Nanjing Agricultural University, Nanjing 210095, China; Department of Biology, Faculty of Science, Wilfrid Laurier University, 75 University Ave W, Waterloo, Ontario N2L 3C5, Canada; College of Horticulture, Nanjing Agricultural University, Nanjing 210095, China; College of Horticulture, Nanjing Agricultural University, Nanjing 210095, China; College of Horticulture, Nanjing Agricultural University, Nanjing 210095, China

## Abstract

Intercropping, a green and sustainable planting pattern, has demonstrated positive effects on plant growth and the soil environment. However, there is currently little research on the influence of intercropping leguminous plants and using them as green manure on the soil environment and tea quality. During the profuse flowering period of Chinese milkvetch, the contents of tea amino acids and soluble sugar in intercropping tea plants with soybean increased by 6.89 and 54.58%. Moreover, there was 27.42% increase in soil ammonium nitrogen and 21.63% increase in available nitrogen. When Chinese milkvetch was returned to soil for 1 month during its profuse flowering period, the soybean and Chinese milkvetch as green manure enhanced tea amino acids and soluble sugar by 9.11 and 33.96%, and soil ammonium nitrogen, nitrate nitrogen and available nitrogen increased by 25.04, 77.84, and 48.90%. Intercropping systems also have positive effects on tea quality components, soil fertility, and soil microbial communities during the profuse flowering period of soybeans and when soybeans with this period were returned to the field for 1 month. Furthermore, the soil fertility index was significantly increased, especially in the intercropping system of tea–soybean–Chinese milkvetch. The soil bacterial community complexity and fungal community interactions were significantly increased. Soil pH, nitrate nitrogen, and available phosphorus were found to be crucial influencing factors on soil microbial communities, specifically bacterial communities. These results highlight the significance of optimizing intercropping systems to improve the soil environment and tea quality components. They also provide a theoretical foundation for promoting the sustainable development of tea plantations.

## Introduction

The tea plant (*Camellia sinensis* L.) thrives in acidic soil and is widely cultivated for its economic value. The tender leaves of tea plants are used to produce tea, which is one of the top three most popular non-alcoholic beverages worldwide [1, 2]. Tea leaves contain a variety of bioactive compounds, including tea polyphenols, catechin, amino acids, caffeine, and soluble sugar. These compounds play a crucial role in determining the unique quality characteristics of tea, such as its color, aroma, and taste [3]. The quality-related metabolites in tea are influenced by various factors, such as tea cultivars, environmental conditions (climate, soil), agronomy practices (intercropping, rotation, no tillage, fertilization), and the tea manufacturing process [4–6]. Intercropping planting patterns have been found to be an effective and sustainable approach in managing tea plantations. Intercropping systems not only help reduce environmental pollution caused by excessive fertilization, but also enhance tea yield and improve tea quality [7, 8]. The soil environment plays a crucial role in the productivity and quality of tea, encompassing soil physical and chemical properties, soil enzymes, and soil microbial communities [9, 10].

Intercropping planting patterns have been found to increase environmental biodiversity, enhance crop quality and yield, improve resource utilization, and reduce the need for chemical fertilizers [7, 11, 12]. The interactions below ground and in the rhizosphere among different plant species are essential for promoting plant growth and metabolism [13]. A large amount of literature has documented the widespread practices of cereal/legume intercropping. Legumes were used in intercropping systems due to their nitrogen fixing capacity, which was very valuable as green manure, especially in cropping systems with chronic nitrogen deficiency [14–16]. Recent studies have examined the practice of intercropping legumes with tea plants during the summer. The intercropping patterns in tea plantation have the ability to alter soil bacterial communities, thereby influencing amino acid metabolism and flavonoid biosynthesis [17–19]. As a result, this practice has the potential to enhance soil nutrient metabolism and improve the quality of tea. Intercropping legumes with non-leguminous species, along with the inoculation of *Rhizobium*, has been shown to have more beneficial effects. The presence of both inoculated rhizobia and native bacteria can alter the bacterial community in the rhizosphere, leading to an enhancement in biological nitrogen fixation and improvement in soil fertility [20]. The structure and activities of the soil microbial community are regulated by the efficiency of soil carbon and nitrogen [21]. Soil microorganisms are major drivers of biogeochemical cycles at the global scale and in the regulation of natural ecosystems [22]. They are closely involved in plant growth, resistance, and resilience [23, 24]. Soil fungal communities are particularly important in regulating ecosystem services related to soil fertility, while the bacterial community is closely connected to plant functional diversity and soil properties [25, 26].

At present, nitrogen fertilizer application increase the tea yield and the amino acids content of the tea leaves. The excessive and long-term use of fertilizer could alter the soil's chemical composition, soil pH, and soil fungal diversity and composition in tea plantations [27]. To alleviate these problems, some studies of intercropping soybean in young tea plantations found that the quality components of tea leaves and soil nutrients were improved. However, the effects of intercropping different leguminous green manure in tea plantations on soil nutrients, microbial communities, and tea quality remain uncertain. In this study, we measured the soil physiochemical properties, soil enzyme activities, and tea quality components, and also analyzed the soil bacterial and fungal community composition using DNA-based sequencing methods (Illumina MiSeq). The results indicated that intercropping with soybean and Chinese milkvetch exertsa large influence of intercropping systems on the composition and diversity of tea plantation soil microbial communities, and significantly improves soil fertility and tea quality.

## Results

### Soil physicochemical properties, enzyme activities, and soil fertility index

Soil pH significantly increased during the profuse flowering period of Chinese milkvetch (period 1) and the profuse flowering period of soybean (period 3) of the tea–soybean–Chinese milkvetch treatment (T1). The contents of soil total nitrogen (TN), total carbon (TC), and soil organic matter (SOM) were significantly increased in intercropping planting systems (*P* < 0.05). Compared with monoculture tea plantations, the levels of available nitrogen (AN), ammonia nitrogen (AMN), and nitrate nitrogen (NN) were lower in the soil of the tea–soybean–Chinese milkvetch system during period 1. However, in the tea–soybean system, these levels increased by 21.63, 27.42, and 7.94%, respectively. The soil's available phosphorus (AP) and available potassium (AK) contents in intercropping planting systems showed significant increases, improving by 492.42 and 41.06% in T1, and by 310.61 and 72.70% in tea–soybean (T2). When Chinese milkvetch in the profuse flowering period was returned to soil as green manure for 1 month (period 2), it was observed that the soil AN, AMN, NN, and AK contents were the highest in the tea–soybean–Chinese milkvetch system. These contents increased by 48.9, 25.04, 77.84, and 10.2% respectively, compared with the monoculture tea plants. During the summer periods (period 3 and period 4), the soil nutrient contents showed a significant increase, except for AP, when using intercropping patterns. Specifically, the content of soil AN, AMN, AP, and AK in the tea–soybean–Chinese milkvetch system was significantly higher than in the tea–soybean system in period 3. In period 4, all these nutrients, except AP, were significantly higher when intercropped soybean was only used as green manure (Table 1).

**Table 1:** Soil physiochemical properties of tea plantations with different treatments.

**Soil property**	**Period 1**			**Period 2**			**Period 3**			**Period 4**		
	**CK**	**T1**	**T2**	**CK**	**T1**	**T2**	**CK**	**T1**	**T2**	**CK**	**T1**	**T2**
pH	5.08 ± 0.02a	5.13 ± 0.01a	4.96 ± 0.04b	4.69 ± 0.01a	4.62 ± 0.03a	4.37 ± 0.13b	4.78 ± 0.02a	4.9 ± 0.07a	4.79 ± 0.03a	4.67 ± 0.07b	4.88 ± 0.03a	4.83 ± 0.03a
Total nitrogen (g/kg)	0.49 ± 0.008c	0.68 ± 0.001b	0.85 ± 0.026a	1.21 ± 0.004c	1.26 ± 0.013b	1.34 ± 0.017a	1.17 ± 0.011b	1.25 ± 0.015a	1.23 ± 0.037ab	1.11 ± 0.012c	1.34 ± 0.03b	1.44 ± 0.014a
Soil available nitrogen (mg/kg)	18.17 ± 0.28b	15.91 ± 0.29c	22.76 ± 0.31a	37.6 ± 0.67b	55.98 ± 0.23a	37.26 ± 0.07b	15.42 ± 0.15b	30.88 ± 0.07a	30.61 ± 0.31a	14.9 ± 0.19c	25.95 ± 0.15b	30.03 ± 0.16a
Ammonium nitrogen (mg/kg)	13.15 ± 0.13b	12.69 ± 0.2b	16.75 ± 0.2a	20.61 ± 0.21b	25.76 ± 0.23a	12.62 ± 0.09c	7.55 ± 0.09c	15.37 ± 0.03a	13.24 ± 0.13b	8.7 ± 0.2b	9.08 ± 0.54ab	9.87 ± 0.09a
Nitrate nitrogen (mg/kg)	5.57 ± 0.16a	3.22 ± 0.12b	6.01 ± 0.21a	16.99 ± 0.49c	30.22 ± 0.13a	24.65 ± 0.15b	7.87 ± 0.24c	15.51 ± 0.09b	17.38 ± 0.24a	6.2 ± 0.08c	16.87 ± 0.27b	20.15 ± 0.16a
Total carbon (g/kg)	4.72 ± 0.13c	5.19 ± 0.01b	6.69 ± 0.07a	10.17 ± 0.25b	10.72 ± 0.01b	12.38 ± 0.08a	11.22 ± 0.02c	12.83 ± 0.13a	11.74 ± 0.05b	11.54 ± 0.08c	13.45 ± 0.07b	13.86 ± 0.03a
Soil organic matter (mg/kg)	8.22 ± 0.23c	9.04 ± 0.02b	11.66 ± 0.13a	17.72 ± 0.44c	18.68 ± 0.02b	21.57 ± 0.14a	19.54 ± 0.03c	22.35 ± 0.23a	20.46 ± 0.09b	20.1 ± 0.14c	23.43 ± 0.12b	24.14 ± 0.05a
Available phosphorus (mg/kg)	0.41 ± 0.09c	2.43 ± 0.24a	1.69 ± 0.19b	5.61 ± 0.27a	4.27 ± 0.27b	4.61 ± 0.19b	9.07 ± 0.94b	13.7 ± 1.4a	6.33 ± 0.11c	10.56 ± 1.02c	26.59 ± 0.55a	19.01 ± 1.1b
Available potassium (mg/kg)	96.49 ± 0.84c	136.12 ± 2.78b	166.65 ± 0.85a	143.85 ± 0.47b	158.54 ± 2.89a	123.16 ± 0.35c	160.97 ± 3.96c	241.04 ± 0.89a	224.2 ± 0.9b	139.69 ± 1.59c	222.5 ± 3.14b	242.78 ± 0.61a
C:N	9.72 ± 0.23a	7.65 ± 0.02b	7.92 ± 0.24b	9.89 ± 0.18a	8.48 ± 0.08c	9.25 ± 0.06b	9.56 ± 0.08b	10.27 ± 0.11a	9.57 ± 0.27b	10.44 ± 0.1a	10.01 ± 0.2b	9.64 ± 0.11b

CK, monoculture tea plants; T1, intercropping tea plants–soybean–Chinese milkvetch; T2, intercropping tea plants–soybean. Lower-case letters indicate significant differences (*P* < 0.05) between different treatments in the same period.

**Figure 1:**
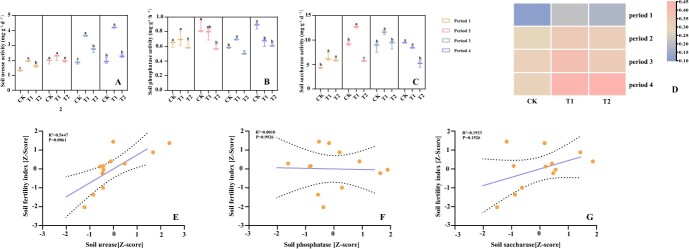
Effects of intercropping legume green manure in tea plantations on soil enzyme activity and average soil fertility index, and regressions between average soil fertility index and soil enzyme activity. **A** Soil urease activity. **B** Soil phosphatase activity. **C** Soil saccharase activity. **D** Soil fertility index. **E** Correlation between soil urease and soil fertility index. **F** Correlation between soil phosphatase and soil fertility index. **G** Correlation between soil saccharase and soil fertility index. Lowercase letters indicated significant differences (*P* < 0.05) between different treatments in the same period.

**Figure 2:**
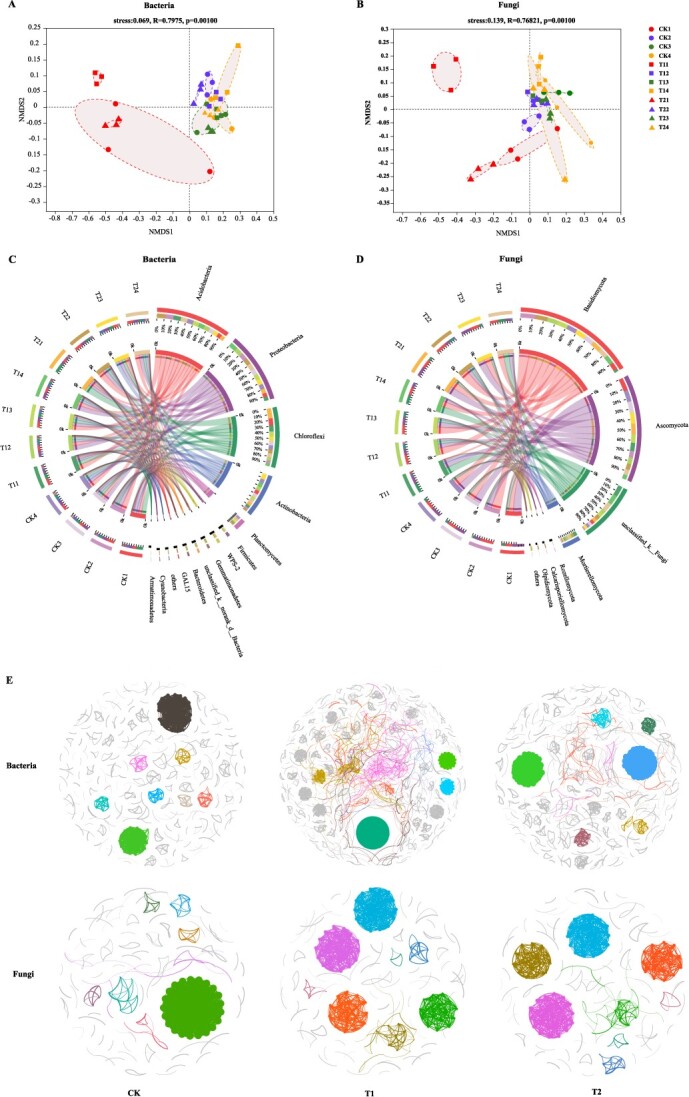
Taxon-specific changes in soil microbial communities for different treatments. **A**, **B** Non-metric multidimensional scaling (NMDS) ordination plot based on the Bray–Curtis distance of samples for the bacterial (**A**) and fungal communities (**B**) in four periods. **C**, **D** Circos sample–species relationship diagram for bacterial taxa (**C**) and fungal taxa (**D**) at the phylum level. The outer ribbon's color indicates the group it belongs to, while the inner ribbon's color represents the species. The length of the ribbon corresponds to the species in the sample, and it also reflects the relative abundance or distribution proportion among the species. **E** Co-occurrence network of the operational taxonomic unit (OTUs) for bacteria and fungi in the intercropping leguminous green manure treatments. The modularity class gives the network nodes different colors. The relationships show substantial (*P* < 0.001) and robust (Spearman's *R* > 0.6) associations. The numbers assigned to each node's edges are reflected in the size of each node.

The soil urease activity of intercropped tea plantations was found to be increased, with the highest activity observed in the tea–soybean–Chinese milkvetch intercropping system (Fig. 1A). The soil phosphatase activity in the tea–soybean–Chinese milkvetch intercropping system was significantly increased over that of monoculture tea plants in period 3 (Fig. 1B). The soil saccharase activity was significantly higher in the intercropping systems during period 1, and it was found to be the highest in the tea–soybean–Chinese milkvetch treatment during periods 2 and 3. Additionally, it was observed that the saccharase activity was increased in the tea–soybean system during period 1 (Fig. 1C). The soil fertility index showed significant increases in intercropping systems of tea–soybean–Chinese milkvetch and tea-soybean and the leguminous plants as green manure in four different periods. Specifically, the soil fertility index increased by 86.46 and 55.63%, 20.87 and 12.88, 22.48 and 4.75%, and 44.32 and 46.85%. Notably, the intercropping pattern of tea–soybean–Chinese milkvetch and these leguminous plants as green manure demonstrated a more pronounced increase in soil fertility during periods 1, 2, and 3 (Fig. 1D). In addition, the soil fertility index was positively correlated with the activities of soil urease (*P* < 0.01) and soil saccharase (Fig. 1E and G).

### Soil microbial diversity and community composition

Compared with monoculture tea plants, the tea–soybean–Chinese milkvetch intercropping system showed an increasing trend in the richness and diversity of the soil bacterial community, except during the profuse flowering period of Chinese milkvetch (period 1) (Supplementary Data Table S1). Additionally, the processing of intercropping leguminous green manures significantly increased the diversity of the soil fungal community (Supplementary Data Table S2). A significant positive correlation was observed between microbial richness and the soil fertility index (*P* < 0.05), while microbial richness showed a negative correlation with soil pH (Supplementary Data Fig. S1A–D). Bacterial community diversity had a significant positive correlation with the soil fertility index, but a negative correlation with soil pH (Supplementary Data Fig. S1E and G). In contrast, the relationships with the fungal community were opposite to those observed with the bacterial community (Supplementary Data Fig. S1F and H).

The community structure of soil bacteria and fungi in tea plantations with intercropping leguminous green manure underwent significant changes during the four periods (*R* = 0.7975, *P* < 0.01; *R* = 0.6821, *P* < 0.001) (Fig. 2A and B). Among the different treatments, the changes in fungal community structure were stronger compared with bacteria in period 1, while the microbial community structure changes were more significant in period 2, as demonstrated by ANOSIM tests using the Bray–Curtis distance (*P* < 0.001) (Supplementary Data Table S3).

The relative abundance of the main bacterial and fungal phylum was >1% in four different periods (Fig. 2C and D). For the tea–soybean–Chinese milkvetch intercropping system, in the profuse flowering period of Chinese milkvetch and soybean (period 1 and 3), the relative abundances of Acidobacteria and Planctomycetes increased, while they were decreased when soybean and Chinese milkvetch were returned to the soil as green manure for 1 month (periods 2 and 4), and this trend was opposite for Proteobacteria. The relative abundance of Gemmatimonadetes increased in the profuse flowering period of Chinese milkvetch and when it was returned to the soil for 1 month (periods 1 and 2). Actinobacteria only increased when Chinese milkvetch was returned to the soil for 1 month (period 2). The relative abundance of Proteobacteria and Chloroflexi in the tea–soybean system significantly increased during the profuse flowering period of Chinese milkvetch (Fig. 2C). The relative abundance of unclassified_k_Fungi and Mortierellomycota increased, while the relative abundance of Ascomycota decreased in the intercropping systems, particularly when tea plants were intercropped with soybean and Chinese milkvetch. The relative abundance of Rozellomycota in the tea–soybean–Chinese milkvetch system was reduced only in the profuse flowering period of Chinese milkvetch (period 1), but it was significantly increased in when soybean and Chinese milkvetch were incorporated into the soil (periods 2 and 4). On the other hand, Basidiomycota relative abundance was increased only during the profuse flowering period of Chinese milkvetch with the tea–soybean intercropping system (Fig. 2D).

### Co-occurrence patterns of soil bacterial and fungal communities

Co**-**occurrence networks were separately generated for soil bacteria and fungi in different intercropping systems, and their associated topological properties were calculated. The intercropping systems exhibited higher modularity and average clustering coefficient in the soil fungal networks compared with the soil bacteria networks (Fig. 2E). In intercropping systems, the average degree and density in the bacterial networks generally increased, whereas in fungal networks they tended to decrease (Supplementary Data Table S4). Both the soil's bacterial and fungal co-occurrence networks indicated that the numbers of links and nodes in the networks generally increased when leguminous green manure was intercropped and used as green manure (Fig. 2E, Supplementary Data Table S4). Analysis of the overall topological properties revealed that all the network structures exhibited scale-free, modular, and non-random characteristics. Furthermore, distinct microbial co-occurrence patterns were observed in different intercropping patterns, with the fungal networks showing a higher level of clustering during the intercropping processing (Fig. 2E).

### Soil environmental factors affecting microbial community structure

**Figure 3:**
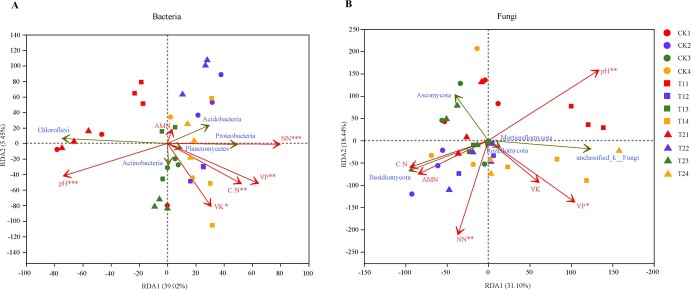
Redundancy analysis showing the correlation between the soil microbial community and soil physicochemical parameters in different treatments for four periods, including (**A**) bacterial phylum-level taxonomy and (**B**) fungal phylum-level taxonomy. *P < 0.05, **P < 0.01, ***P < 0.001. Sample groups under different environments or conditions are represented by points of different colors or shapes. Red arrows denote quantitative environmental parameters, while green arrows signify species. The extent of the environmental factor's influence on the species data is shown by the length of the arrow. Positive correlation is represented by an acute angle, while negative correlation is represented by an obtuse angle, and a straight angle indicates no correlation at all. The relative influence of environmental factors on the distribution of the sample community is represented by the distance from the projection point from the sample point to the arrow of the quantitative environmental factor and the origin.

The environmental attribution was limited to the first and second sorting axes, according to the two canonical axes. Redundancy analysis (RDA) accounted for 39.02 and 31.10% of the variation in the bacterial and fungal structures, respectively. Among the treatments, soil physicochemical factors significantly affected the soil microbial communities (Fig. 3). In relation to the bacterial phyla, soil pH was obviously and negatively correlated with the relative abundance of Proteobacteria, Acidobacteria, and Planctomycetes. However, there was a strong and positive correlation between soil pH and the relative abundance of Chloroflexi. The relationships between these bacteria and soil NN, AP, AK, and C:N ratios were found to be opposite to the soil pH value. The phylum Actinobacteria was positively correlated with soil pH value, AP, AK, and C:N ratios (Fig. 3A). In terms of fungal phyla, Basidiomycota showed a significant positive correlation with NN, while it was significantly and negatively correlated with soil pH value. The Ascomycota displayed a negative correlation with soil NN, AP, and AK (Fig. 3B).

### Formation and correlation analysis of tea quality components

The quality components of fresh tea leaves were found to be closely related to tea quality. In the profuse flowering period of Chinese milkvetch, the contents of epigallocatechin gallate (EGCG), epigallocatechin gallate (ECG), amino acids, and soluble sugar in the tea–soybean system were higher compared with the control group (CK). Specifically, there was an increase of 2.80% in EGCG, 22.70% in ECG, 6.89% in amino acids, and a significant increase of 54.58% in soluble sugar. When Chinese milkvetch was incorporated into the soil for 1 month, the tea–soybean–Chinese milkvetch treatment showed higher levels of EGCG, ECG, amino acids, and soluble sugar compared with the monoculture tea plants (CK). Specifically, the levels increased by 4.59, 10.60, 9.11, and 33.96% respectively. In the profuse flowering period of soybean and when soybean was incorporated into the soil for 1 month (periods 3 and 4), intercropping leguminous green manure significantly promoted the formation of EGCG, ECG, amino acids, and soluble sugar in tea plant leaves. This effect was particularly evident when tea plants were intercropped with soybean and Chinese milkvetch, as well as when the two leguminous plants were both incorporated into the soil as green manure. It should be noted that the content of tea polyphenols in the tea–soybean–Chinese milkvetch system was lower than in the monoculture tea plants (*P* < 0.05) (Table 2).

The formation of amino acids was significantly and positively correlated with the soil pH value and AMN content, but negatively correlated with soil carbon, TN, AP, and AK. With the exception of soil pH, the soluble sugar content in tea plants demonstrated a significant positive connection with soil physicochemical parameters. With the increase of soil AN and AMN, the EGCG level dramatically increased while the caffeine content decreased. Tea polyphenols and amino acids were higher when the soil AP decreased. Additionally, we observed that tea catechins, EGCG, and amino acids were lower when there was more AK remaining in the soil. It is worth noting that almost all soil physicochemical properties showed a positive correlation (Supplementary Data Fig. S2).

**Table 2:** Quality components of fresh tea leaves in different treatments.

**Tea plant property**	**Period 1**			**Period 2**			**Period 3**			**Period 4**		
	**CK**	**T1**	**T2**	**CK**	**T1**	**T2**	**CK**	**T1**	**T2**	**CK**	**T1**	**T2**
Tea polyphenols (%)	20.78 ± 0.64a	19.47 ± 0.79b	20.59 ± 0.41ab	20.39 ± 0.41a	20.56 ± 0.58a	19.49 ± 0.67a	21.19 ± 0.74b	19.52 ± 0.53c	22.63 ± 0.36a	18.74 ± 0.45a	17.53 ± 0.07b	17.88 ± 0.12b
Catechins (%)	13.9 ± 0.55a	13.13 ± 0.76a	14.2 ± 0.26a	13.55 ± 0.41a	13.84 ± 0.32a	13.33 ± 0.39a	11.29 ± 1.21a	12.52 ± 0.13a	11.6 ± 0.5a	13.9 ± 0.2a	13.03 ± 0.77a	13.42 ± 0.18a
EC (%)	0.34 ± 0.069a	0.37 ± 0.024a	0.21 ± 0.01b	0.39 ± 0.027a	0.31 ± 0.039b	0.40 ± 0.019a	1.26 ± 0.07a	1.23 ± 0.03a	1.20 ± 0.03a	0.48 ± 0.01a	0.36 ± 0.04b	0.38 ± 0.01b
EGC (%)	0.84 ± 0.048b	0.93 ± 0.01a	0.92 ± 0.012a	0.85 ± 0.009b	0.88 ± 0.006a	0.86 ± 0.01b	1.07 ± 0.08a	1.17 ± 0.009a	1.10 ± 0.03a	0.89 ± 0.003a	0.84 ± 0.02c	0.87 ± 0.006b
ECG (%)	1.23 ± 0.06b	1.56 ± 0.04a	1.51 ± 0.05a	1.22 ± 0.10b	1.35 ± 0.03a	1.32 ± 0.01ab	1.31 ± 0.13b	1.59 ± 0.05a	1.36 ± 0.17ab	1.26 ± 0.01b	1.46 ± 0.02a	1.41 ± 0.044a
EGCG (%)	9.62 ± 0.14ab	8.13 ± 1.34b	9.89 ± 0.24a	9.57 ± 0.13ab	10.01 ± 0.23a	9.42 ± 0.35b	6.59 ± 1.06b	7.5 ± 0.1a	6.79 ± 0.33a	9.19 ± 0.09a	8.81 ± 0.47a	9.01 ± 0.13a
Amino acids (%)	3.95 ± 0.05b	4.01 ± 0.17ab	4.22 ± 0.04a	3.1 ± 0.1b	3.38 ± 0.08a	3.11 ± 0.04b	2.22 ± 0.03b	2.43 ± 0.07a	2.3 ± 0.07b	2.24 ± 0.02c	2.88 ± 0.05a	2.68 ± 0.09b
Caffeine (%)	2.93 ± 0.26a	3.48 ± 0.01a	3.31 ± 0.23a	2.87 ± 0.34a	3.07 ± 0.13a	3.32 ± 0.15a	3.91 ± 0.11a	3.76 ± 0.06ab	3.6 ± 0.08b	3.59 ± 0.16a	3.36 ± 0.05a	3.59 ± 0.05a
Soluble sugar (%)	3.43 ± 0.24b	3.45 ± 0.57b	5.3 ± 0.02a	5.4 ± 0.32b	7.23 ± 0.53a	5.68 ± 0.13b	4.82 ± 0.13b	5.22 ± 0.18a	5.18 ± 0.11a	5.21 ± 0.39b	6.22 ± 0.45a	5.8 ± 0.17ab

CK, monoculture tea plants; T1, intercropping tea plants–soybean–Chinese milkvetch; T2, intercropping tea plants–soybean. EC indicates epigallocatechin; EGC indicates epigallocatechin. Lower-case letters indicate significant differences (*P* < 0.05) between different treatments in the same period.

## Discussion

### Effects of intercropping leguminous green manure on soil nutrients

Many studies have consistently shown that plant species diversity is essential for fostering soil fertility and facilitating the uptake of soil nutrients [28, 29]. In this study, the soil physicochemical properties were found to be significantly increased by intercropping leguminous green manures in period 1, except for soil AMN and NN. Among the different intercropping combinations, the tea plant–soybean intercropping treatment showed the most pronounced effects. Warm temperatures enhanced microbial activity, accelerated the breakdown of organic matter and released organic nitrogen during the plant growing season [30, 31]. The study found that intercropping leguminous green manure significantly increasedsoil nutrient properties，especially when the profuse flowering period of soybean and soybeans were incorporated into the soil (Table 1). This improvement is achieved through niche complementarity between grain legumes and non-legumes, which helps in improving total nitrogen capture [32]. The results of the study demonstrated that intercropping patterns and the leguminous plants as green manures influenced the conversion efficiencies of essential nutrients. Moreover, it was observed that different plant species had distinct impacts on soil properties. The presence of a high diversity of plant species could enhance the variety of litter quality and types, thereby promoting the diversity of mutualistic microfauna and other animal groups [33]. Our study showed that the processing of intercropping leguminous green manure in tea plantations increased the soil fertility index. Previous studies have suggested that legumes can enhance soil fertility by utilizing fixed nitrogen as complementary resources [34]. Therefore, intercropping leguminous plants and these plants as green manure has been found to both have a positive impact on the physicochemical properties and significantly increase soil fertility in tea plantations. This effect was particularly noticeable when tea plants were intercropped with soybeans and Chinese milkvetch.

### Influence of intercropping leguminous green manure in shaping soil microbial community diversity

The changes in above-ground vegetation species diversity also contribute to variations in soil factors, potentially leading to differences in bacterial and fungal community diversity [35]. Our study showed that soil pH was significantly negatively correlated with bacterial diversity, while no significant correlation was observed with fungal diversity. A previous study indicated that soil pH has a strong influence on the bacterial community and can be attributed to the narrow pH ranges that favor optimal bacterial growth, whereas fungal growth seems to be less affected by soil pH [36]. The soil fertility index proved to be a crucial factor in predicting the diversity of microbial communities. We observed a significant positive correlation between the soil fertility index and bacterial diversity, but no significant correlation with fungal diversity. The correlation between soil fertility and fungal biodiversity may be weakened in ecosystems that have experienced anthropogenic pressure, such as tea fields [37]. However, the impact of soil fertility on fungal diversity cannot be overlooked, considering the crucial role fungal communities play in ecological functions such as the recycling and mobilization of mineral nutrients [25]. Therefore, changes in soil pH and soil fertility are closely related to soil microbial communities in tea plantations intercropped with leguminous green manure.

### Taxon-specific changes in soil microbial communities for intercropping leguminous green manure in tea plantations

At the bacterial phylum level, Acidobacteria, Proteobacteria, Chloroflexi, and Actinobacteria were identified as the predominant phyla in tea plantations. These phyla are commonly found in bacterial communities in soils worldwide [38]. In the intercropping system of tea plant–soybean–Chinese milkvetch, the relative abundance of Acidobacteria and Planctomycetes increased during the profuse flowering period of Chinese milkvetch and soybean. But the relative abundance of Acidobacteria and Planctomycetes decreased when Chinese milkvetch and soybean were incorporated into the soil as green manure. Additionally, the relative abundance changes of Proteobacteria were found to be opposite to that of Acidobacteria and Planctomycetes in four different periods (Fig. 2C). Previous studies have shown that different subdivisions of Acidobacteria have varying correlations with soil nutrients and play a role in regulating ecological function [39]. Proteobacteria are considered copiotrophic organisms that contribute to biocrust formation and soil stability [40]. Ascomycota and Basidiomycota are particularly prominent fungi in soils as they play an important role in carbon cycling through the degradation of organic substances. Mortierellomycota and Rozellomycota are key rhizosphere microbiomes and are induced to promote plant nutrient absorption by rhizobia and arbuscular mycorrhizae [41]. Therefore, different planting patterns of intercropped leguminous green manure have been found to have varying effects on the soil microbiome. These patterns are also significant factors that influence the structure and composition of microbial communities. Ultimately, these factors impact the recycling of soil carbon, nitrogen, and phosphorus nutrients.

The co-occurrence network analysis revealed that intercropping legumes and incorporating them into the soil as green manure leads to a higher level of bacterial community organization and a greater potential for bacterial species interactions. Small-world networks, which are characterized by short path lengths, are known for their ability to quickly respond to disturbances in ecosystems [42]. This study also observed that the fungal co-occurrence network in intercropping systems exhibited higher values for the modularity index and average clustering coefficient (Supplementary Data Table S4). Previous research has shown that a modularity index >0.4 indicates the presence of typical module structures and strong resistance to environmental changes [43]. Therefore, intercropping legumes and utilizing them as green manure led to an augmentation in the complexity of the bacterial community and enhanced interactions within the fungal community. These outcomes align with earlier studies on the enhancement of the ginseng rhizosphere. [44].

### Effects on the formation of tea key quality components in tea leaves

Intercropping, especially with legumes, is a productive and sustainable system that can enhance plant growth and improve soil quality [45, 46]. This study observed that intercropping leguminous green manure significantly promoted the formation of soluble sugar, amino acids, and EGCG, especially the intercropping system of tea–soybean–Chinese milkvetch. These changes in the quality components were closely related to tea quality, nutritional benefits, and health benefits [47]. The high contents of amino acids and soluble sugar in tea could enhance the umami flavor of tea infusion, thereby improving tea quality [3]. Our research results indicated that intercropping leguminous green manure has the potential to enhance the quality of tea. We observed significant variations in tea quality based on different intercropping planting patterns and the use of various intercropping plants as green manure. Furthermore, our study reveals a strong correlation between the quality components of tea plants and soil nutrients, which is consistent with previous research [48]. Supplementary Data Fig. S2 presents a noteworthy positive correlation between the formation of soluble sugars in tea leaves and soil nutrient parameters. Additionally, there was a significant positive correlation between the synthesis of amino acids in tea plants and soil pH and ammonia nitrogen. Moreover, the level of EGCG in tea plant leaves exhibited a certain degree of inertia with soil AN and ammonia nitrogen. Consequently, this study emphasized the strong correlation between soil physical and chemical parameters and the formation of essential quality components in tea.

In addition, the significance of soil pH and nitrogen as influential factors in shaping soil microbial communities has been previously established [49]. In tea plantations, other studies have also found a negative relationship between the relative abundance of Acidobacteria and soil pH [50], which aligns with the findings of our study. The Proteobacteria phylum, which consists of various microorganisms, plays a crucial role in plant growth and symbiotic nitrogen fixation, thereby enhancing the availability of nitrogen in the soil [51]. The Chloroflexi were commonly found to be involved in photoautotrophic carbon fixation [52]. These studies suggested that variations in the bacterial community could be influenced by edaphic factors, particularly carbon and nitrogen. Soil pH and NN were identified as the major factors affecting fungal communities. Bacterial communities were more strongly impacted by soil NN compared with fungi, as nitrogen concentration played a crucial role for bacteria and fungi. The response of microorganisms to nitrogen may be influenced by different forms of nitrogen [53]. Previous studies have demonstrated that in environments with high levels of soil nutrients or under nitrogen enrichment, there is an increase in the abundance of Ascomycota while the abundance of Basidiomycota decreases [54, 55]. The variation in fungal compositions could be influenced by plant diversity, soil environmental temperature, and humidity [56]. Consequently, intercropping leguminous green manure and returning them to the soil may be the primary explanation for changes in soil microbial communitie. The species and quantities of intercropping legumes play a crucial role in this process, ultimately affecting the formation of soil nutrients and their utilization by tea plants. The correlation analysis between soil physicochemical parameters and key quality components of tea in Supplementary Data Fig. S2 indicated that variations in soil environmental factors directly impact the growth and development of tea plants, consequently influencing the formation of key quality components in tea leaves.

## Conclusion

**Figure 4:**
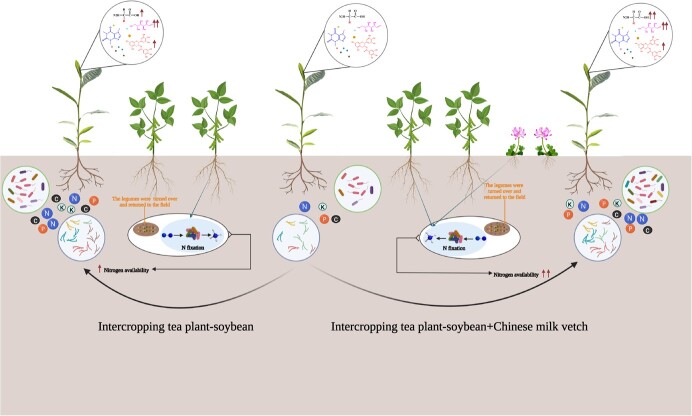
Intercropping leguminous green manure created positive above- and below-ground legacies that influenced the tea quality components and soil nutrition, as well as changing microbial community composition and structure. The figure was created using BioRender (https://biorender.com/).

In this study, intercropping leguminous green manure in tea plantations could effectively regulate the diversity and richness of the soil microbial community, increase fungal community diversity and interactions, and enhance the complexity of the soil bacterial community and soil fertility, particularly in the intercropping system of tea plant–soybean–Chinese milkvetch. The microbes involved in accelerating nutrient cycling were closely associated with the soil physicochemical parameters and had a significant impact on soil pH, NN, and AP content (Fig. 4). This study also confirmed that intercropping leguminous green manure in tea plantations increased the levels of important quality-related components in fresh tea leaves. These components include EGCG, amino acids, and soluble sugar, and their levels were found to be enhanced during the process of intercropping leguminous plants and using them as green manure. These results provide a basis for further investigation into the soil environment of tea plantations intercropped with leguminous green manure and the underlying mechanisms of tea plant growth and metabolism. Additionally, intercropping tea plants with soybean and Chinese milkvetch and using them as green manure might be a promising strategy to more effectively promote sustainable production in tea plantations.

## Materials and methods

### Site selection and soil sampling

The tea plantation used in this study was provided by Nanjing Botea Agricultural Science and Technology Co., Ltd. It is located in Hengxi town, Nanjing City, Jiangsu Province (31°37′–32°07′ N, 118°28′–119°06′ E). The tea plantation received fertilization twice a year, with 300 kg/ha of urea in February and 750 kg/ha of compound fertilizers in October. The experimental design consisted of the following groups: monoculture tea plants as a control, tea plants intercropped with soybean and Chinese milkvetch (referred to as T1 or tea–soybean–Chinese milkvetch), where soybean was planted in the summer and Chinese milkvetch in the winter, and tea plants intercropped with soybean (referred to as T2 or tea–soybean), where soybean was planted in the summer. The soybean was inoculated with USDA110 (*Bradyrhizobium japonicum*) and the Chinese milkvetch with Mh93 (*Mesorhizobium huakuii*).

The experiment was initiated in October 2017. The total area of the experimental site was 650 m^2^. Within this area, there were three experimental plots for each treatment. Each plot had a size of 50 m^2^, calculated by multiplying the length of 25 m^2^ by the width of 2 m^2^. To separate each experimental treatment, there were two rows of tea plants, and the leguminous green manure was sown between the rows of tea plants as a practice. Prior to planting the green manure in intercrops, it was necessary to lightly prune the tea trees, particularly the side branches. Additionally, the pruned tea branches were carefully removed from the tea rows. In mid-May of each year, the soybean seeds were inoculated with rhizobium bacteria and sown in holes between two rows of tea plants. Three to five seeds were placed in each hole. The rhizobium milkvetch seeds that had been inoculated were broadcast in mid-October annually. The recommended dosage of green manure seeds was 30 kg/ha, and the sowing hole or trench was at a depth of 5–10 cm. Finally, the seeds were covered with a thin layer of soil and irrigated once.

Soil samples and tea plant leaves were collected four times in 2019. The collection periods were as follows: 17 March 2019 (period 1): during the profuse flowering period of Chinese milkvetch; 16 April 2019 (period 2): Chinese milkvetch in profuse flowering period was returned to the soil as green manure for 1 month; 25 July 2019 (period 3): during the profuse flowering period of soybean; and 25 August 2019 (period 4): soybean in the profuse flowering period was returned to the soil as green manure for 1 month. During the profuse flowering period, the average biomass of soybeans and milkvetch was 19.2 and 1.2 g/plant, respectively. Soil samples were collected at a depth of 0–15 cm after remove the covering on the soil using the S-shaped sampling method of soil transverse sampling. The collected soil samples were sieved, homogenized, and then subdivided for analysis. They were either stored at −80°C for microbial community analysis or at 4°C, or air-dried and ground for chemical property profiling. A bud and the first and second leaves from the top bud of the tea plants were carefully picked, pan-fired, and dried for the analysis of tea quality components. All samples of this study were three biological replicates.

### Analysis of tea quality components

The catechins and caffeine were analyzed following national standards (GB/T 8313-2008) using UPLC (Ultimate 3000, Thermo, USA) [57]. Tea polyphenols were measured using the foline–phenol method as described in GB/T 8313-2008. The total free amino acids were determined with the ninhydrin colorimetry method by GB/T 8314-2013. Soluble sugars were quantified using the anthrone colorimetric method described by Redillas *et al*. [58].

### Soil physicochemical properties and enzyme activity

Soil pH was measured with a pH meter (FE28, Mettler Toledo, Switzerland) in a suspension with soil to water ratio of 1:2.5. The total carbon and total nitrogen in soil were quantified by dry combustion using an automated TC/TN analyzer (Multi EA 5000, Jean, Germany). Soil AMN and NN were measured using a continuous-flow analytical system (Seal Auto Analyzer AA3, Norderstedt, Germany). Soil organic matter was determined using the K_2_Cr_2_O_7_-H_2_SO_4_ oxidation method [59]. Soil AP was extracted using 0.03 M NH_4_F–0.025 M HCl and measured using molybdenum-blue colorimetry, and soil AK was extracted using 1 M NH4Ac (pH = 7.0) and detected by inductively coupled plasma emission spectrometry (Optima 8000, Perkin Elmer, USA) [60]. Soil enzyme activity was measured with the *p*-nitrophenyl-phosphate method, the sodium phenol-sodium hypochlorite colorimetric method, and the 3,5-dinitrosalicylic acid colorimetric specific terms [61, 62].

### DNA extraction, PCR amplification, and sequencing analysis

A 0.5 g soil sample was taken and DNA was extracted using the MoBIO PowerSoil® DNA isolation kit from Mo Bio Laboratories in Carlsbad, CA, USA. The quality of the DNA was assessed through agarose gel electrophoresis and quantified with a UV spectrophotometer. The DNA was then divided into aliquots and stored in a -20°C refrigerator for future sequencing.

To investigate the soil environmental microbial community, we collaborated with Shanghai Majorbio Bio-pharm Technology (Shanghai, China) to perform a series of techniques. For the amplification of bacterial 16S rRNA, we used the primer pairs 515-F (5′ GTG CCA GCM GCC GCG GTA A 3′) and 907-R (5′ CCGTCAATTCMTTTRAGTTT 3′). Similarly, for the amplification of the fungal ITS region, we used the primer pairs ITS1F (5′ CTT GGT CAT TTA GAG GAA GTA A 3′) and ITS2R (5′ GCT GCG TTC TTC ATC GAT GC 3′). PCR amplification was carried out using Transtar FastPfu DNA Polymerase (AP221-02, TransGen) on an ABI GeneAmp^®^ 9700. The purified amplicons were then pooled in equal amounts and subjected to paired-end 2 × 250 bp sequencing on the Illumina MiSeq platform (San Diego, CA, USA).

The paired-end reads obtained from Illumina sequencing are first spliced based on the overlap relationship. Simultaneously, the sequence quality was controlled and filtered. After distinguishing the samples, OTU cluster analysis and species taxonomy analysis were performed. Multiple diversity index analyses was conducted based on the OTUs. Additionally, sequencing depth detection was performed. Statistical analysis of community structure at each classification level was carried out based on taxonomic information. Building upon the previous analysis, a series of in-depth statistical and visual analyses, such as multivariate analysis and significance testing, were conducted on the community composition and phylogenetic information of multiple samples.

### Statistical analyses

The statistical analysis was conducted using SPSS 22.0 and significance was determined by Duncan’s test (*P* < 0.05). Soil quality indicators were transformed into scores (0–1) using linear and non-linear scoring functions. These scores were then combined into soil quality indices using principal component analysis (PCA) to identify critical parameters [63, 64]. Standardized PCA of all (untransformed) data that showed significant differences between treatments was performed using SPSS 22.0 [63, 65]. Detailed scoring values and weights were assigned to the selected soil fertility parameters (Supplementary Data Table S5). Following this, the soil fertility index was calculated using the following equation:

$$ \mathrm{Soil}\ \mathrm{fertility}\ \mathrm{index}=\sum\limits_{i=1}^{n} Wi\ast Si $$

where *W* is the weight coefficient of each parameter, *S* is the indicator score and *n* is the number of parameters [66]. Using the program R (R Core Team, 2018), we performed non-metric multidimensional scaling analysis (NMDS) based on the operational taxonomic unit (OTU) level to assess the similarity patterns in the composition of the microbial community (Bray–Curtis similarity). We performed co-occurrence network analysis based on Spearman's coefficient to explore the relationship and interactions between different microbial species. Network graphs were calculated and visualized using the igraph R package and Gephi [67]. Environmental factors were screened using the variance inflation factor (VIF), and those with a VIF value >10 were removed to ensure low collinearity (Supplementary Data Table S6). Finally, we analyzed the relationship between soil microbial community structure and each affecting factor using redundancy analysis and variation partitioning.

## Supplementary Material

Web_Material_uhae018

## Data Availability

All relevant data in this study are provided in the article and its supplementary files. The raw sequences of the 16S rRNA gene and ITS were submitted to the NCBI Sequence Read Archive under BioProject accession number PRJNA857577.
