# Supplementary material for: Leguminous green manure intercropping changes the soil microbial community and increases soil nutrients and key quality components of tea leaves
Source: Hortic Res. 2024 Jan 17;11(3):uhae018. doi: 10.1093/hr/uhae018 (PMC10967690; doi:10.1093/hr/uhae018)
Supplement: Web_Material_uhae018 [file web_material_uhae018.zip › Supplementary file.docx]

Table S1 Soil bacterial diversity and richness estimators

| Different period | Chao1 estimator | | | Shannon index | | |
| --- | --- | --- | --- | --- | --- | --- |
|  | CK | T1 | T2 | CK | T1 | T2 |
| Period 1 | 1837.75±199.33a | 1714.07±111.84a | 1985.27±131.69a | 5.34±0.25a | 5.41±0.09a | 5.39±0.13a |
| Period 2 | 2350.15±88.11a | 2386.77±144.55a | 2117.2±53.86b | 6.09±0.1a | 6.15±0.11a | 5.83±0.12b |
| Period 3 | 2061.28±194.27a | 2265.92±42.15a | 1816.37±60.99b | 5.98±0.12a | 5.99±0.09a | 5.85±0.04a |
| Period 4 | 1927.28±123.14b | 2274±64.41a | 2410.29±167.35a | 5.76±0.22b | 5.94±0.25a | 5.99±0.23a |

Note: CK indicates monoculture tea plants, T1 indicates intercropping tea plants–soybean + Chinese milk vetch (Tea–soybean–Chinese milk vetch), and T2 indicates intercropping tea plants–soybean (Tea–soybean), lowercase letters indicate significant differences (*P* < 0.05) between different treatments in the same period

Table S2 Soil fungal diversity and richness estimators

| Different period | Chao1 estimator | | | Shannon index | | |
| --- | --- | --- | --- | --- | --- | --- |
|  | CK | T1 | T2 | CK | T1 | T2 |
| Period 1 | 333.28±24.01a | 254.52±40.54b | 345.09±12.81a | 3.34±0.41b | 3.65±0.07a | 3.75±0.51a |
| Period 2 | 721.84±5.41a | 789.14±63.25a | 556.03±51.13b | 2.98±0.33b | 3.54±0.22a | 3.22±0.53a |
| Period 3 | 524.42±70.98a | 578.01±19.36a | 481±42.74b | 2.82±0.51b | 3.4±0.16a | 3.28±0.1a |
| Period 4 | 493.38±64.02b | 600.56±98.88a | 592.77±59.09a | 2.5±0.47b | 2.94±0.37a | 3.04±0.45a |

Note: CK indicates monoculture tea plants, T1 indicates intercropping tea plants–soybean + Chinese milk vetch (Tea–soybean–Chinese milk vetch), and T2 indicates intercropping tea plants–soybean (Tea–soybean), lowercase letters indicate significant differences (*P* < 0.05) between different treatments in the same period

Table S3 The ANOSIM analysis for soil bacteria and fungi communities in different periods

| Different period | Bacteria | | Fungi | |
| --- | --- | --- | --- | --- |
|  | R | P | R | P |
| Period 1 | 0.6543 | 0.001 | 0.9671 | 0.001 |
| Period 2 | 1 | 0.001 | 0.893 | 0.001 |
| Period 3 | 0.5062 | 0.001 | 0.5638 | 0.001 |
| Period 4 | 0.5556 | 0.001 | 0.4156 | 0.003 |

Table S4 Soil bacterial and fungal network properties at the different intercropping patterns

| Network Parameters | Bacteria | | | Fungi | | |
| --- | --- | --- | --- | --- | --- | --- |
|  | CK | T1 | T2 | CK | T1 | T2 |
| Nodes | 565 | 1335 | 830 | 247 | 299 | 256 |
| Links | 1300 | 10403 | 4385 | 1233 | 1041 | 932 |
| Network diameter | 4 | 29 | 9 | 7 | 7 | 8 |
| Modularity | 0.798 | 0.422 | 0.689 | 0.293 | 0.795 | 0.816 |
| Average clustering coefficient | 0.87 | 0.719 | 0.814 | 0.855 | 0.864 | 0.9 |
| Average path length | 1.127 | 7.094 | 1.775 | 1.32 | 1.268 | 1.497 |
| Average degree | 4.602 | 15.585 | 10.566 | 9.984 | 6.963 | 7.281 |
| Density | 0.008 | 0.012 | 0.013 | 0.041 | 0.023 | 0.029 |

Table S5 Scoring function value and the weight assigned to selected soil fertility parameters

| Parameters | Scoring curve types ^a^ | Lower threshold (x1) | Upper threshold (x2) | Weight |
| --- | --- | --- | --- | --- |
| Total nitrogen (g/kg) | S | 0.5 | 2 | 0.284 |
| Soil available nitrogen(mg/kg) | S | 30 | 150 | 0.275 |
| Soil organic matter (mg/kg) | S | 6 | 40 | 0.271 |
| Available phosphorus(mg/kg) | S | 3 | 40 | 0.072 |
| Available potassium (mg/kg) | S | 30 | 200 | 0.098 |

a: The equation for the scoring curve is as follows:

$$f\left( x \right)=0.1+\frac{0.9\left( x-x1 \right)}{(x2-x1)}$$

Table S6 VIF variance inflation factor for screening

|  | AMN | NN | VP | VK | pH | C:N |
| --- | --- | --- | --- | --- | --- | --- |
| VIF | 333.28±24.01a | 254.52±40.54b | 345.09±12.81a | 3.34±0.41b | 3.65±0.07a | 3.75±0.51a |


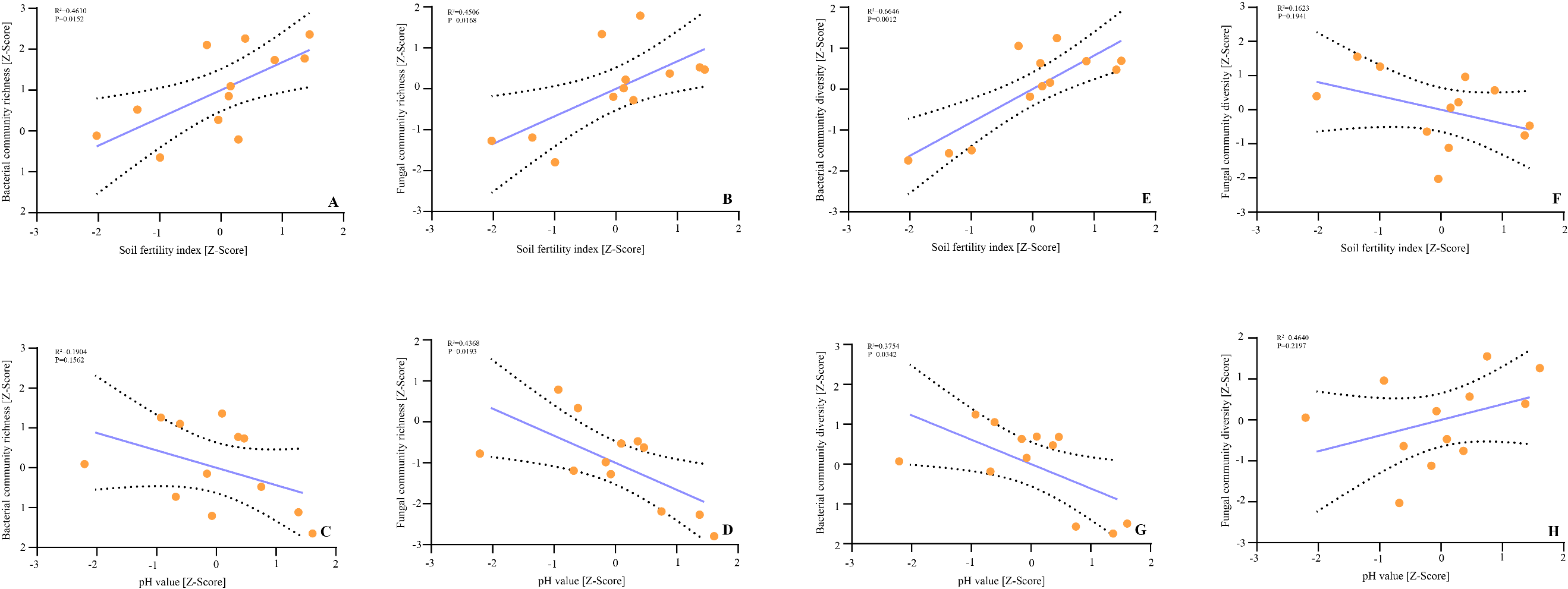


**Figure S1**

Regressions between the soil fertility index, soil pH and soil microbial community diversity and richness


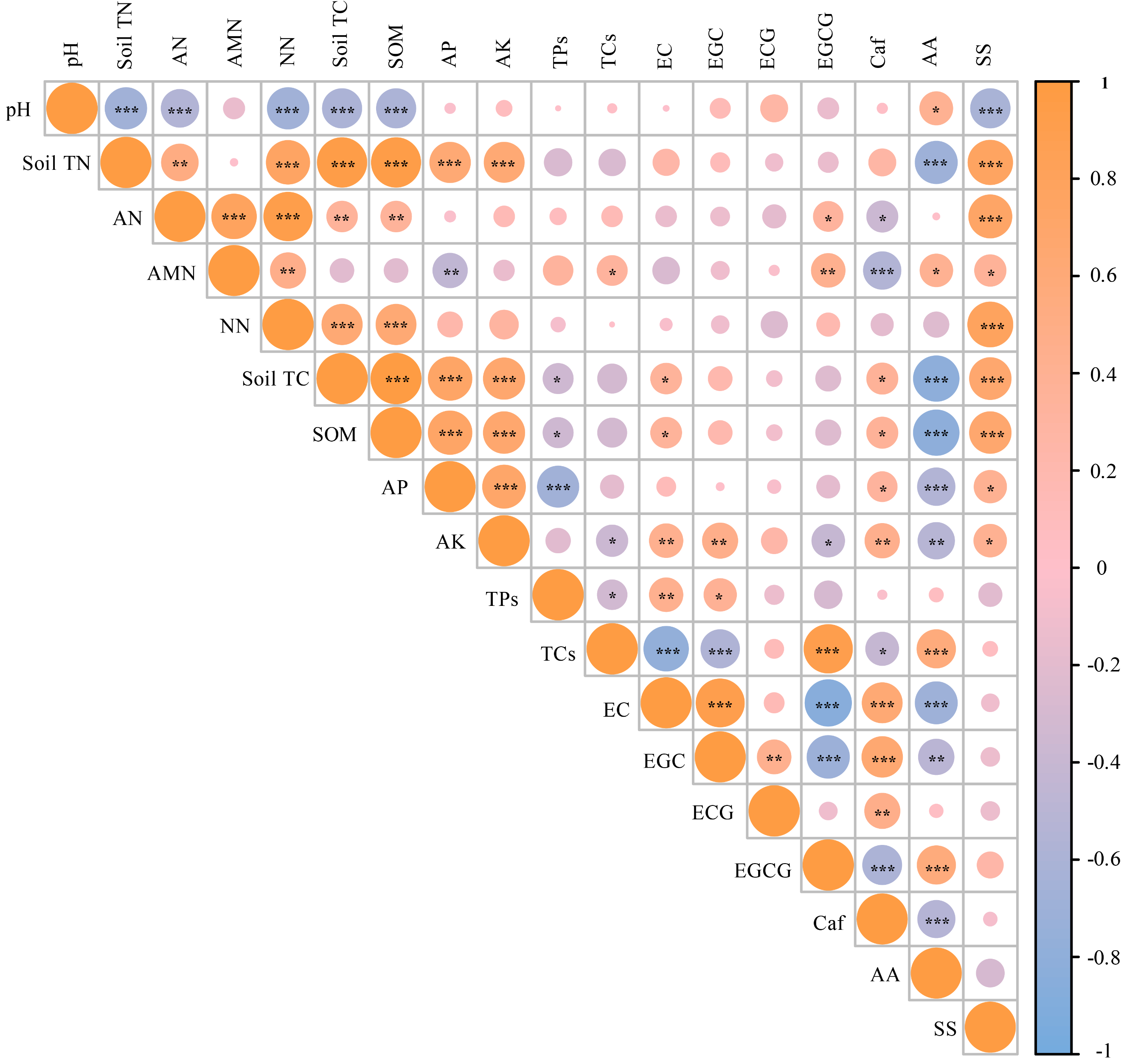


**Figure S2**

The correlation between the soil physicochemical properties and tea quality components

* indicated *P*<0.05. ** indicated *P*<0.01, *** indicated *P*<0.001
